# “It’s not just the residents who need to be motivated for activity”: a qualitative study of the perspectives of staff on providing activity support for people with psychiatric disabilities in supported housing in Sweden

**DOI:** 10.3389/fpsyt.2023.1322859

**Published:** 2024-01-05

**Authors:** Rosita Brolin, Carina Tjörnstrand, Mette Friis, Elisabeth Argentzell, Ulrika Bejerholm, Mona Eklund, David Brunt

**Affiliations:** ^1^School of Health and Caring Sciences, Linnaeus University, Växjö, Sweden; ^2^Department of Health Sciences/Mental Health, Activity and Participation, Lund University, Lund, Sweden

**Keywords:** community psychiatry, human activities, people with disabilities, staff attitudes, staff development, social support

## Abstract

**Background:**

The goals for staff in Supported Housing for people with psychiatric disabilities include helping to develop the residents’ independence and self-confidence in activities. However, staff have expressed frustration about providing this type of support when motivating residents to engage in meaningful activities and also about the difficulty in finding suitable levels of independence within a housing setting with limitations.

**Objective:**

The aim is to explore the views and experiences of housing staff in Supported Housing on how they can stimulate and support engagement in activities for people with psychiatric disabilities.

**Methods:**

Twenty-six members of staff from 20 supported housing units in 10 municipalities in Sweden were interviewed in five focus groups. A semi-structured interview guide was used, and the transcribed material was analyzed using qualitative content analysis.

**Results:**

Three main categories emerged from the analysis: *Multi-faceted factors influencing the staff’s provision of activity support*, *Staff’s approach for supporting activities*, and *Staff’s struggles to develop their work*. Obstacles to participating in activities in the community were identified. Many contrasting factors were found, such as spontaneous or structured activities and individual or group activities, which affected the staff’s ability to motivate to activity.

**Conclusion:**

A broad approach encompassing in-house training including a focus on values, recruitment policies, staff supervision and interventions focusing on both residents and staff are ways to support staff in motivating residents toward being more active within Supported Housing.

## Introduction

Good housing, and the security that is entailed in having such housing, has been said to be a pre-requisite for people with mental illness to be able to be active in daily life and to create an occupational identity (1, 2, 3). Other factors that can contribute to this include having the right amount of activities and the right variation between different forms of activity (4) as well as having a balanced pattern of individually meaningful activities (5). However, it has been shown that people with psychiatric disabilities living in Supported Housing (SH) have a low level of activity and spend a great deal of their time alone in their apartment (6). Moreover, the residents themselves struggle to find a balance between how much support they need and how much support is to be given by the staff and they are frustrated about not receiving the right type of support (3, 6).

Congregate living solutions, which were mainly introduced in Sweden in the 1990s and later termed supported housing, met the basic needs of people with mental illness who had often spent many years in hospital settings and who were deemed unable to live alone with outreach support. These needs included safety, privacy, health, an individualized lifestyle, as well as a place to return to, stability and shelter (7). The most common category of staff in SH in Sweden is nursing assistants (8). They generally receive some form of in-house training, which often has a more clinical approach with a focus on understanding psychiatric disabilities. There is a general understanding that the goal for the staff in SH should be to provide a secure and safe housing, and to help the residents in their rehabilitation pathway, which is related to having meaningful activities and also to help them to gain greater independence and self-confidence in engaging in activities on their own (9). A study in the United Kingdom showed that effective practice in SH was aided by supporting independence and creating a good relationship where the residents felt they were acknowledged and appreciated and experienced that the support was tailored to the individual. Such individual support was seen as important for the residents to feel engaged, both socially and as a part of the surrounding community (9), which is in line with a systematic review and narrative synthesis of what matters the most for a better life situation according to people with psychiatric disabilities themselves (10). However, there appear to be difficulties in the provision of such support due to the available levels of resources and opportunities in SH units as well as the knowledge and prevailing attitudes. Moreover, recent studies in Sweden show that suitable levels of independence and security within the SH service have not always been experienced by the residents (3, 11). Furthermore, the residents’ self-determination has been found to be restricted in a manner reminiscent of psychiatric institutions (12).

The staff in SH in a study in Sweden have expressed frustration and spoken of difficulties in engaging individuals in targeted goal-directed activities (13), thus indicating a need for guidance from professionals who are specialized in rehabilitation. Lindström et al. (14) showed that individuals living in SH were dependent on staff for support when pursuing new activities and that a rehabilitative approach was important for the individual to realize an agentic identity. They noted in the residents’ narratives that staff who did not have a rehabilitative approach more often created dependency. This could be, for example, by helping and doing the cleaning for the individual instead of supporting his/her independence to perform the task on his/her own, as also shown in Tjörnstrand et al. (6). On the other hand, staff with a rehabilitative approach are more often perceived as having ‘their hands behind their backs’ when working, guiding the residents to carry out activities on their own. Furthermore, earlier research (3, 6) has indicated that regular staff support based on the residents’ individual needs motivated engagement in activities from the residents’ perspective. Other factors also seen as important for remotivating to engagement in activity were providing individually adjusted stimuli in the physical and social environment. However, motivating and engaging factors in the housing context have not been studied from a staff perspective.

There are a number of implementation components that may either facilitate or hinder the implementation of activity engagement. These include a lack of funding, which can affect the possibilities for employing staff with specialized knowledge, and also create a lack of opportunities for development and advancing a career within these services, which in turn can make it difficult to retain staff. This is problematical as a positive work environment is important for the staff and maintaining staff continuity helps to create security for the residents (15). Moreover, profound and responsive relationships with the residents take time to develop and are core factors for supporting a rehabilitative approach and forming individualized support as well as for how staff perceive their opportunities to support the residents to be active to the extent they desire (3, 16). Furthermore, the staff need to feel they have the skills and the opportunity to tailor this kind of support and to motivate toward engagement in activity for the residents. More knowledge is thus needed about the staff’s experience of both their opportunities and their difficulties in providing beneficial support to the residents of SH in order to be able to design and fashion the support needed.

### Aim

The aim of the current study is thus to explore the experiences of housing staff in SH on how they can motivate and support engagement in activities for people with psychiatric disabilities.

## Materials and methods

### Sample

The study is part of a larger project investigating SH in Sweden to develop adequate support for this population (17). The design and content of SH units vary greatly between municipalities and the aim was to strategically search for variations in environmental aspects such as staff structure, indoor and outdoor areas, and access to rural and urban community environment. The conditions may also differ in different regions of Sweden and units across regions in the southern more populated half of the country were thus sought. A total of 10 municipalities agreed to take part: five from districts in a major city and five from smaller cities. The unit managers then received both oral and written information to convey to the staff. Twenty-six members of staff from 20 different housing units expressed an interest in participating in the focus groups and gave written consent. Some of the units were located in the outskirts of smaller cities, closer to rural outdoor activities, while others were located in a large city within an urban environment. Twenty of the participants were female. Twenty-three were nursing assistants, often with some form of in-house training focusing on mental illness and psychiatric disabilities, two were supervisors and one was a coordinator. The five focus groups consisted of staff from several different units, and the size of the groups varied between three and seven.

### Settings

The SH facilities in Sweden most commonly consist of fully fitted apartments including a bathroom, a sleeping space, and an eating space. All the SH in the study contained 5–12 apartments and had a staffed common area with a sitting room, an eating area. The facilities for group activities varied in size depending on the layout of the building and the staff support was in most cases 24 h per day.

The SH residents had been assessed by a social worker as unable to live in ordinary housing with outreach support and in need of continuous staff support. They received counseling and treatment either from a mental health service team or from a community mental health centre, and nurses and occupational therapists visited the SH when called for. Some of the SH tried to employ staff with a university degree, such as social workers and behavioral scientists, although the absence of a career path increased the staff turnover for those with a higher education. The support given to the residents by the staff varied from psychological support to helping with everyday tasks such as medical appointments, shopping, cleaning, and leisure activities. The policy for food provision varied from not providing any cooked food to one meal per day or all meals. The staff supported the residents in cooking or reheating ready-prepared food. Most of the units were administered by municipalities and one was privately run.

### Procedure

The focus groups were conducted by an occupational therapist (author MF) familiar with the SH environment. A psychologist with knowledge from facilitating focus groups acted as a second group leader who summarized the main focus of the group at the end of each interview. One of the authors (CT) participated in the first two interviews to provide support when needed. The questions were derived by the research group based on their collective knowledge from SH and informal in-depth interviews with two occupational therapists who had worked in SH. The interviews had a dual purpose, to gain a better understanding of the SH context from the occupational therapist perspective and to derive questions that would be important to ask the staff. The questions that were derived in this way were semi-structured with the aim of stimulating discussions in the groups. Three main content areas were presented at the beginning of each focus group by the leader; (i) the residents’ everyday activities - how they can be stimulated, (ii) environmental impact/social relations, (iii) the road to better health and quality of life among the residents. Each content area had support questions that could guide the discussions such as: ‘What support needs do you think the residents require for being able to carry out the activities they wish to do?’, ‘What access is there today in housing to promote social relations inside and outside the housing unit to facilitate activity participation?’, and ‘What is needed to make it better?’. The leader ended each session by summarizing what had been said to ascertain whether the information had been correctly understood. The interviews, which were recorded and transcribed verbatim, lasted approximately 2 hours with a short break after about 1 hour.

### Ethical considerations

The Regional Ethical Review Board approved this study (LU 2013/456). All the participants were informed that confidentiality was assured and that they could withdraw their participation at any time.

### Analysis

Two of the authors listened to the recorded focus group interviews several times in order to gain an overall view of the content. The recordings were then transcribed verbatim. Pauses and laughter were noted, as well as when several of the participants were talking at the same time. The transcribed material was analyzed with qualitative content analysis (18). Each of the five transcribed focus group interviews was analyzed as a separate unit of analysis. Two authors (RB and MF) first performed a manifest analysis where the material was divided between these two. This was due to administrative and practical considerations, such as the length of employment in the project. The analysis process was conducted on paper and started by identifying meaning units that were relevant to the aim of the study. All meaning units were then condensed in the next step and a first coding was conducted. Author RB coded three of the analysis units, while author MF coded two. These two authors then compared their coding, discussed similarities and differences, and made a joint decision regarding the designations of the codes as well as on which meaning units should belong to each code. Author RB then carried out an integrated analysis on a more latent level and formulated preliminary categories and sub-categories. A third author (DB) read through the preliminary analysis and RB and DB came to an agreement about the categorization that they both considered to best represent the data. The analysis resulted in three main categories, with three subcategories in each.

## Results

The results are presented in Table 1, and in the following text.

**Table 1:** Categories and subcategories.

Main categories	Subcategories
Multi-faceted factors influencing the staff’s provision of activity support	*The settings* *The residents’ personal preferences and social situation* *Obstacles to participation in activities in the community.*
Staff’s approach for supporting activities	*Focusing on the residents and their individual development* *Utilizing rewards as an incentive* *Balancing structure and continuity* versus *spontaneity*
Staff’s struggles to develop their work	*Difficulties staff face in their everyday work* *Organizational and structural disincentives, and opportunities* *Needs for supervision, education, and greater knowledge about activities*

### Multi-faceted factors influencing the staff’s provision of activity support

The staff described *the settings*; *the residents’ social situation and personal preferences*; and *obstacles to participation in activities in the community.*

#### The settings

There were two main settings for the residents’ activities according to the staff: the SH unit and the community. Many activities were carried out alone in the residents’ own apartments, although watching TV with other residents and staff to have some company was popular. Communal activities, such as cooking, film evenings and board games, were often organized by the staff. The balcony was a favorite place where spontaneous conversations could occur, “they can go out onto the balcony for a cigarette …. that balcony is popular and that’s where they can meet and talk a little.” The staff also spoke of difficulties in trying to get the residents to be involved in activities in the community but perceived that the residents felt comfortable and safe in the unit, where they could easily withdraw to their own apartment if needed. A limited range of activities in the local municipality also entailed a challenge for the staff if a resident had tried the few available activities without finding anything of interest.

#### Residents’ personal preferences and social situation

The staff noted that activities outside the SH that demanded pre-registration and/or attendance control were less popular among the residents who preferred more spontaneous activities such as going to the cinema, a café, or a restaurant. They also appreciated concerts, football matches and journeys. The staff surmised that these types of activities helped the residents “to feel for a while … that they are just like everyone else.” The staff also reasoned that the residents preferred activities that were perceived as meaningful. One such activity was gardening, which led to being outdoors and seeing plants grow, bloom and bear fruit. Pets were another meaningful activity, which entailed the residents giving them daily walks, love and attention, and sufficient food. One unit had rabbits in outdoor cages, which also led to more contact with neighbors and people passing by, “a lot of people who walk here […] stop and …and many children, yes, the rabbits have been very popular.” Playing table tennis, billiards, and boule and relaxing in the sauna were further examples of meaningful activities.

Most of the residents had an extremely limited social network, for example, celebrating birthdays and festivities with staff and co-residents rather than their own families. The age distribution among the residents was broad and uneven, which made it a challenge to design and offer activities appealing to everyone. The staff experienced that younger people who had moved into the SH units often expressed dissatisfaction with existing activities more suited to older residents. They want to “do normal things, just like others in that age like doing.” The staff felt they had run out of ideas of activities that might appeal to the younger ones and often only suggested “sport” and “badminton.”

#### Obstacles to participation in activities in the community

The staff identified some situations that constituted a disincentive or deterrent from performing activities outside the safe home environment. For example, the cost of organized activities in the community caused many of the residents to refrain from participation due to their economic situation. Furthermore, fear, anxiety, or obsession made some of the residents choose to stay at home, and transportation (e.g., bus travel) to the activity was a hindrance, while physical obstacles could make it difficult to participate for older residents. The residents’ medication could also be an impediment with high dosages leading to fatigue, or low dosages entailing the resident being too worried and anxious to participate. The staff also reported that the residents did not always get on with each other and disagreements could occur, making it difficult to motivate them for group activities; and splitting the group into two parts could sometimes facilitate participation.

#### Staff’s approach for supporting activities

Several of the staff had received in-house method training, such as Needs Assessment, an Independent Life, Motivation interviews and Case Management. Widely varying approaches were used to motivate and support the residents in activity participation. These approaches emerged in the analysis: *Focusing on the residents and their individual development*; *Using rewards as an incentive*; *Balancing structure and continuity* versus *spontaneity*.

#### Focusing on the residents and their individual development

The staff tried to adapt their actions to the unique needs of each resident and usually found activities to do together with the resident in his/her apartment when the latter did not want to go out, such as cooking together, watching movies, reading newspapers or books, and discussing what they had read. They emphasized the importance of understanding situations from the residents’ perspective: “they live so close together, sometimes you have to have respect for that […] they have not chosen to live together with the other residents.” Despite this focus on individual development the staff also found they were tempted to take over tasks to do them quicker or get a better result and had to remind themselves of the objective of supporting increased independence.

The staff found it easy to motivate residents to engage in gardening and the care of pets. The residents were pleased to mow the lawn, and care for the vegetables and flowers. They gladly fed the pets, cuddled with them, or repaired rabbit cages etc. The response they received from working with plants, pets and staff made them feel needed and important. On the other hand, the staff found it difficult to support the residents in getting to know their surroundings and making contacts with organizations in the community that could potentially provide meaningful activities. The staff maintained that the residents found it “stressful and difficult” and had heard them say that “they will not be seen properly,” thus wanting the staff with them for support.

#### Utilizing rewards as an incentive

A majority of the staff agreed that rewards and incentives were a major motivational factor in stimulating the residents to be more active. The staff found it easiest to motivate residents to participate in activities that were free of charge, or if they got something to eat, for example, during movie evenings and “cosy Friday evenings” with snacks, “those who we do not see so much otherwise, they’ll come when it’s time for crisps.” Similarly, the residents could be motivated to plan meals, go shopping, bake, or cook, if this entailed them having a free dinner or coffee and a sandwich. The residents could receive a small sum of activity remuneration for taking responsibility for everyday tasks in the housing unit, such as carrying out household waste, doing the washing up, and cleaning common areas, “you do not want to work for nothing, you are not a volunteer.”

The staff reflected on the fact that their discussions about their motivational efforts showed similarities to those that parents have about motivating their teenagers to help at home: “…it will not work to just say a yes, but you can get…we can go out and get a coffee or you can have something else free of charge, but no, it’s got to be money, otherwise they will not do it.” Another form of reward could include doing a pleasant activity together with a member of staff, for example, one resident “is very interested in billiards and we have combined it with the housing support, so it’s called ´cleaning and billiards’ and on those days when he’s in form then he’ll do some of the cleaning together with us and then we play billiards, a good combination with a little reward.”

#### Balancing structure and continuity versus spontaneity

Important tools for stimulating activities according to the staff were having structure and continuity that generated a sense of security for the residents. Allocating a key worker with a responsibility to activate “their” resident, where the latter was free to choose what to do in the time designated for them was one of the staff’s approaches. A noticeboard with information about the staff’s working hours for the current week helped to provide the structure, which was appreciated by the residents. Although if someone was sick or had a day off or if it was during the summer holidays then they were “very anxious about checking on the noticeboard… who is working and if there’s a stand-in then they are a bit apprehensive.” A noticeboard was also used for information on the week’s planned activities, including such tasks as watering the flowers and feeding the pets. Other structuring tools were action plans and implementation plans for individual residents, with goals and sub-goals, which were reviewed and updated periodically.

Residents tended to refrain from organized group activities, and the staff had to be flexible in their approach to motivate their participation. Explaining that it is better to join one of the 10 sessions than not join at all was one method, while talking about the activity and then say “I’ll wait outside” was another. Each resident was thus given some time and space to prepare, and this was described as more effective than “trying to cajole them and say ´come on now` and harping on about it.”

However, structure and continuity were not always good for all the residents, planned activities could cause anxiety for some. A spontaneous suggestion for an activity such as baking, doing a jigsaw puzzle, playing cards, board games or video games could be sufficient to attract residents to participate and sometimes be more successful than scheduled activities. Furthermore, a spontaneous and relaxed interaction between the residents and the staff could be achieved by the latter just being present in the communal area every afternoon (and not in the office).

Motivational interviews (MI) were sometimes used, where the staff tried to lead the conversation toward what the residents used to do earlier in their lives, and which activities they had experienced as meaningful, rewarding, or fun. This method was also used for motivating residents for everyday activities, such as having a shower. Instead of saying that the resident has not showered for 2 weeks, “Say … Do you remember how pleasant it felt when you’d just had a shower?’… ‘Yes, I remember’… ‘then you liked being clean and fresh’ and then they start to think… it gives them the idea that they are the ones who decide… you sow a little seed, an idea, and then you can go on with it.”

#### Staff’s struggles to develop their work

The staff initially found it difficult to respond to the question about their own needs for further education, supervision, and training to be able to stimulate the residents to be more active and needed to be prompted by the interviewer. They then responded more directly about their needs and three subcategories emerged in the analysis: *Difficulties they face in their everyday work*, *Organizational and structural disincentives, and opportunities* and finally, the staff’s view of their own *Needs for supervision, education, and greater knowledge of activities*.

#### Difficulties staff face in their everyday work

The staff spoke of a frustration when planning activities, “you can decide on a Monday about doing something the day after, but when Tuesday comes the resident does not feel well and then it’s canceled […] it can be very frustrating,” and the residents’ limited economy could prevent them from participating in activities that cost money. They tried to compensate this by arranging opportunities for activity in the house or the garden. “We arranged a little gym here and that was fun for a couple of weeks but then the residents got tired of it.”

A challenge they faced was to balance meeting the individual’s needs for support, while stimulating his/her development toward independence and also protecting them from factors that can adversely affect their health and well-being. This resulted in the staff facing daily ethical dilemmas where they must prioritize one of the roles. The staff perceived this as a difficult balancing act, maintaining that there was a risk of prioritizing the protective role to the extent that it would be like a parenting role.

Another dilemma faced by the staff was when “Family days” were organized and the residents’ relatives and friends were invited to have a cup of coffee or tea at the housing unit. Not all the residents received a visit from their relatives and the staff discussed about how to act to reduce the suffering for those individuals, without affecting the fellow residents in a negative way. Not arranging family days, because one of the residents does not have any relatives to invite “felt as though we would disregard all the others, that they could not have their relatives there.”

Some focus group participants wanted to discuss values since they felt quite alone in their view of considering the residents as equal human beings. They also expressed a frustration about the staff easily becoming stuck in old routines. Two fractions appeared within the staff group: those who prioritized cleaning and similar tasks and those who prioritized doing activities together with the residents. One participant maintained that it is important to motivate the staff to create opportunities for activities for the residents and said, “It’s not just the residents who need to be motivated for activity.”

#### Organizational and structural disincentives and opportunities

Organizational and structural factors that could be promoting or obstructive were discussed in all five groups. These factors included human resources, leadership, schedules, geographical differences, and local conditions. The size and layout of the SH units varied thus providing differing opportunities for activities. A lack of space was a constant concern, “we wish we could have a hobby room where we could sit and paint or do jigsaws or whatever,” while other units had larger communal areas, and access to a garden. Similarly, the availability of activities in the community and financial resources varied between the municipalities. Having a kitty to cover the staff’s costs when accompanying the residents to a café was available in some municipalities but not in others. Access to a minibus facilitated transportation to activities but several units shared one with other units leading to difficulties in planning activities. Using the minibus entailed a small cost and some residents did not want to or were unable to pay their share. “Not all of them have that money” and “there are many of them who cannot use public transport […] because of their social phobia.”

The staffing levels also varied between the SH units, but the staff in municipally run units agreed that there was no time to accompany one or two residents on an evening activity in the local area due to the need for extra staff, which was not allowed. It could even be difficult to get sufficient staff resources daytime because there was no budget for stand-ins in case of sick leave among the ordinary staff. Those working in a privately-run SH unit spoke of having much better opportunities to accompany residents to activities, “then I can change my working hours […] a lot of these opportunities came when the private company took over.”

#### Needs for supervision, education and greater knowledge about activities

Some participants spontaneously maintained that the staff group worked well together and that they did not have any need for supervision or education. One claimed that they were young and good at examining themselves and their work and would thus not get stuck in old ways of working and did not need supervision. The importance of supervision for “older” staff groups was, however, emphasized by some younger staff members. After further deliberation, several participants stated that it might be valuable to get some education about, for example: “disabilities”; “technical aids”; “motivational conversation”; “residents who isolate themselves… what do you do?”; or “about hoarders, to get to know more about working with them.” Attending courses was described as rewarding and inspiring, as it provided them with opportunities to exchange experiences with others. The importance of renewing working methods was emphasized, since the target group had changed from mainly consisting of older people with a long experience of life in an institution, to a mixed group with also younger people with differing needs together with the older ones.

The participants concluded that it was often easiest to just work as they had “always done” but reflected that having supervision could perhaps help them to find ways to inspire and motivate the residents for activities. Furthermore, some situations could generate the need for someone to talk to, for example, when a new resident moves in, or in connection with stressful events. A need was also expressed about having “a knowledge bank” with information about available activities for the residents at the SH and in the community.

## Discussion

The present study helped to explore how staff may motivate and support engagement in activities for the residents in SH. Multiple factors across the identified categories and subcategories were related to staff’s provision of support, the approach they used when doing so, and their struggles to develop their work along the way. The following discussion will consider the results of the analysis in three main sections; contrasting factors and the environment as disincentives for the residents and obstacles for the staff in motivating engagement in meaningful activities, incentives for the residents and facilitators for the staff’s support for the residents’ engagement in activities, and the staff’s need for supervision and training.

There were contrasting factors that impacted the staff’s possibilities to provide support. The staff spoke of the opportunities for activities in the common area of the housing unit that often featured the provision of food and drink and thereby creating possibilities for social interaction. On the other hand, they also spoke of difficulties in motivating residents for more structured group activities, and often needed to deal with contrasting factors as presented above. It also differed as to where residents preferred to engage in activities, e.g., in the housing unit or outside in the community. Belonging to different age spans (older and younger age) was also decisive when choosing certain activities. Furthermore, it also varied as to what extent residents appreciated spontaneous activities or those with more, structure and routines. What was greatly appreciated was the ability to choose an activity and then engage in it together with a staff member. Unfortunately the insufficient staffing levels in the SH made it impossible to carry out such activities on a regular basis, which could lead to frustration on both sides. The findings indicate a dilemma for the staff in attempting to balance these contrasting aspects in their desire to motivate activity among the residents.

One contrasting aspect that creates difficulties for the staff is found in the intrinsic nature of congregate housing for people with psychiatric disabilities (2). The inherent protective nature of these housing settings helps to meet the basic needs of the residents but can at the same time be prohibitive in terms of social interaction and activity participation. This finding was further corroborated in a review by Jose et al. (19) showing that residents with medium levels of support participated in more activities, and in the community, than those in SH with a high level of support. Facility size (20), higher levels of choice (21), and less complex needs and fewer symptoms in accommodation with medium support (15) have been presented as contributory factors to this difference. To conclude, the congregate nature of the residents’ living situation, with heterogeneous groups of people with a wide range of ages, interests and personalities being forced to live together, and where psychiatric disability is their only common denominator, can generate obstacles and complications for the staff in their attempts to support the residents’ engagement in activities. At the same time, the staff took notice of the residents’ opposition to being part of an “institution.” This feature may be viewed as a strength where the residents show a will to become more autonomous. For residents to feel self-determination and power, even if it is about being able to refrain from participating in group activities can also be seen as positive and something for the staff to recognize and pay attention to, which is in line with a previous study by Brolin et al. (11). Other research further emphasizes the important element of self-acceptance and connectedness to other peers (22), when individuals engage in meaningful social activities and finding friendship with other residents (3).

Rewards of various types were seen by the staff as a major motivational factor in stimulating the residents to engage in different activities. Food and drink were attractive to the residents, which concurs with the findings of Brolin et al. (12). Monetary rewards, free food or not having to pay for an activity were important incentives for the residents to engage more in activities, while also facilitating for the staff in their motivational work. Receiving a small remuneration for performing household duties was one example of such a reward, which highlights another factor, the poor financial situation of the residents that was an obstacle for the residents’ participation in activities outside the housing unit. A clear link has been established between poverty and severe mental illness (23), including being more likely to belong to a lower socioeconomic group. Poverty may also constitute an obstacle for recovery (24), which was evident in the present study where the staff reported financial constraints as a reason for the residents abstaining from activities, otherwise found vital for personal recovery (25, 26). An innovative intervention study carried out by Topor and Ljungkvist (27), where people with severe mental illness were given a financial contribution of 500 Swedish crowns (approx. 53 EUR) per month for 9 months to facilitate social activity, reported improved living conditions and social interactions for the participants. Such an initiative would be beneficial for SH residents providing an important incentive for increasing their activities and social interaction outside the ´safe haven` (28) of the SH setting, thus also boosting their journey toward individual and meaningful goals.

The study showed that the staff faced daily ethical dilemmas in terms of prioritizing motivation and support for the residents’ independence and self-determination or protecting them from factors that could adversely affect their health and well-being. This concurs with the findings of Sandhu et al. (6), who described it in terms of a “common tension between providing safe and supportive living environments, whilst also promoting independence and facilitating rehabilitative change” (p. 1), and with Fossey et al. (29) where residents described the same tension. Supported housing units with their built environment with integrated common areas supervised by on-site professionals 24/7 have been perceived as mini institutions (30). This atmosphere taken together with the difficulties as discussed above also impacts the staff, who sometimes find themselves routinely following old patterns, expressed in the interviews as working as “we have always done.” Staff competencies and their needs for support and supervision to be better equipped to meet this dilemma are thus important to discuss.

Two interesting aspects concerning competence and the need for supervision emerged in the focus groups The first relates to the difficulty many of the staff had in answering a question about their own needs of further education, revealing this was not a common discussion among the staff, perhaps indicating a lack of awareness of or unwillingness for self-examination. The second concerned a polarization within one of the groups between those who wanted to discuss values, saw the residents as equals and prioritized spending time together with them and those who continued with their usual routines and prioritized cleaning and similar chores. A related aspect, which some of the staff found difficult to handle, was the temptation to take over tasks to do them quicker or better, while also realizing that they needed to suppress such impulses to support the residents’ development and independence.

The contrasts noted above, rooted in the institution-like setting and the need to balance an individual approach within a congregate housing unit, make the staff’s assignment to a particularly demanding one, which was corroborated in Lindström’s study (13), where the staff spoke of their frustration about it. Two questions are generated by this: Do the staff have sufficient competence/education to perform this difficult task? And do they receive sufficient support/supervision? Most of the staff interviewed in the present study were nursing assistants, which as stated above is the most common category of staff in SH in Sweden (8). This can be seen as a relatively low level of educational and improvements should thus be made. So, what can be done in order for them to be better equipped to face the difficulties in activating SH residents? Recruiting trained peer support workers and staff with a college education in rehabilitation, social work, occupational therapy, or behavioral science is one way to begin to meet this challenge, albeit a long-term strategy. A commitment to develop career opportunities to retain trained staff is another. Staff supervision in groups is also a way to raise the level of competence among the staff. Furthermore, various types of in-house training were spoken of in the focus groups. The importance of such support has been advocated by Bitter et al. (31) who conclude that the staff in SH need constant support for their development and tailored team-based training linked to the difficulties they face.

So, what should training contain to further the staff’s ability to support the residents in SH settings toward engagement in activities? A greater focus on the residents’ abilities and capacity to change and have personal goals, which is inherent in a rehabilitative perspective, is needed to counteract the institutional nature of SH. Killaspy et al. (15) found that residents in SH with a greater rehabilitative focus increased their participation in leisure and social activities. A number of authors have also highlighted the lack of occupational therapy expertise in terms of activity stimulation in these settings (13, 14, 32). Argentzell et al. (3) maintained that occupational-based interventions that correspond to the residents’ long term goals are warranted in order to facilitate needs for meaningful activities, and Killaspy et al. (15) concluded that gaining skills in rehabilitation practice was key for staff working in this context. However, a systematic review of the efficacy of training for housing staff revealed that there was limited support for a consistent effect of training on staff or service user outcomes. The authors recommended training as constituting one component of a broader approach to service transformation (33). A broader approach should thus also include recruitment of staff with a higher level of education, supervision as well as in-house training. Employing trained peer support workers could also help as a tipping point in a care-giving culture, in order to make the care provided more focused on individual and meaningful goals (22). A further element consisting of interventions involving both staff and residents in a SH unit with the potential of reinforcing the staff in stimulating engagement in activities is recommended. Two different occupational therapist-led interventions, Everyday Life Rehabilitation (13) and Active in my Home (17), both with a rehabilitation focus on individual and meaningful activity goals, are examples of this.

What was striking in the current study was, however, that the staff were divided into two camps. While one side was keen to involve the residents in all kinds of activities where it was possible for these to participate and get something meaningful out of it, the other side often sought “an easy way out,” performed most activities by themselves and preferred not to socialize with residents. Thus, when it comes to support for activity, staff in some of the SH did not fully fulfil their duties and appeared not to be aware of it. This indicates that education where also values pertaining to rights and so-called occupational justice (34) should also be part of the staff training.

## Discussion of method

### Strengths

A study’s trustworthiness can be discussed in terms of its credibility, dependability, transferability and confirmability (35). The credibility was strengthened by using focus groups, which provided rich data and constituted an immediate members check as participants from the same SH validated each other’s stories. Mixing some of the groups with participants from different SH settings was also beneficial in this validation process.

Dependability, which focuses on consistency and whether the findings are repeatable, was strengthened by the use of a second focus group leader who summarized the main focus of the group after each data collection. This allowed the group participants to clarify or bring other subjects to light that they felt to be important as well as ascertaining whether the information had been correctly understood. Moreover, it constituted a second direct members’ check after each interview. Dependability was also strengthened by the use of the coding procedure, in which one author independent from the data collection, coded three of the five analysis units, while another author coded two. They compared their coding, discussed similarities and differences, made joint decisions regarding the coding, and then involved a third author in their continued analysis work. Their differing perspectives contributed to confirmability, which concerns the neutrality of the researcher. The immediate members’ checks and the thorough audit trail explaining the data collection and procedures in the method section, together with the important feedback from the other researchers in the research group, strengthened the study’s dependability.

### Limitations

There were, however, some limitations to this study. Mixed focus groups with participants from different SH settings could have been used to an even greater extent than was actually done in the study but which was limited due to time and organizational factors at the SH units. The dependability of the study could have been further strengthened if both coding authors had coded all five focus group interviews and then compared their codings. Unfortunately, this was not possible, due to administrative and practical reasons, such as employment time in the project. Finally, transferability is limited to similar settings and societies, but is always up to the reader to assess.

## Conclusions/implications for practice

The interviews with the staff in SH provided a deeper understanding of SH as a phenomenon in the Swedish context, particularly the staff’s need for help to meet the difficulties in supporting engagement in meaningful activities among the residents. The results present examples of both what staff saw as functioning methods and as difficulties when providing activity opportunities to the residents in SH and, as well as the balancing act they described between their protective role and showing respect for the residents’ own decisions as adults and stimulating greater independence. A broad approach encompassing in-house training, including a focus on values as maintained above, recruitment policies, staff supervision and interventions focusing on both residents and staff are ways to support staff in the activity support to residents in SH.

### Suggestions for future studies

The present study is part of a larger project that has included an intervention aimed at stimulating the residents to be more active in their own home (17). A study that focuses on further identifying what is needed for staff in SH to be successful in motivating the residents to engage in activities would be a complement to this project. This could be followed by an intervention containing, for example, group supervision for the staff, workshops where both staff and residents participate or a tailored team-based education. The intervention should then be evaluated. A study that explores the values held by staff in SH is also warranted based on the results of the study.

## Data availability statement

The original contributions presented in the study are included in the article/supplementary materials, further inquiries can be directed to the corresponding author/s.

## Ethics statement

The studies involving humans were approved by the Regional Ethical Review Board, Lund University, Sweden (LU 2013/456). The studies were conducted in accordance with the local legislation and institutional requirements. The participants provided their written informed consent to participate in this study.

## Author contributions

RB: Formal analysis, Methodology, Writing – review & editing. CT: Conceptualization, Data curation, Methodology, Writing – review & editing. MF: Data curation, Formal analysis, Writing – review & editing. EA: Conceptualization, Investigation, Project administration, Writing – review & editing. UB: Conceptualization, Investigation, Project administration, Writing – review & editing. ME: Conceptualization, Funding acquisition, Investigation, Methodology, Project administration, Writing – review & editing. DB: Conceptualization, Formal analysis, Writing – original draft.

## References

[1] Kirsh B. Gewurtz R. Bakewell R. Singer B. Badsha M. Giles N. (2009). Critical characteristics of supported housing: findings from the literature, residents and service providers. Available at: http://www.wellesleyinstitute.com/wp-content/uploads/2011/11/Critical_Characteristics_of_Supported_Housing_0.pdf

[2] Krotofil J McPherson P Killaspy H. Service user experiences of specialist mental health supported accommodation: a systematic review of qualitative studies and narrative synthesis. Health Soc Care Community. (2018) 26:787–800. doi: 10.1111/hsc.12570, PMID: 29609195

[3] Argentzell E Tjörnstrand C Brunt D Eklund M Bejerholm U. Opportunities and barriers for occupational engagement among residents in supported housing. Scand J Occup Ther. (2023) 30:125–35. doi: 10.1080/11038128.2022.2141315, PMID: 36345116

[4] Wagman P Håkansson C Björklund A. Occupational balance as used in occupational therapy: a concept analysis. Scand J Occup Ther. (2012) 19:322–7. doi: 10.3109/11038128.2011.596219 21780985

[5] Eklund M Orban K Argentzell E Bejerholm U Tjornstrand C Erlandsson LK. The linkage between patterns of daily occupations and occupational balance: applications within occupational science and occupational therapy practice. Scand J Occup Ther. (2017) 24:41–56. doi: 10.1080/11038128.2016.1224271, PMID: 27575654

[6] Tjörnstrand C Eklund M Bejerholm U Argentzell E Brunt D. A day in the life of people with severe mental illness living in supported housing. BMC Psychiatry. (2020) 20:508. doi: 10.1186/s12888-020-02896-3, PMID: 33059664 PMC7559196

[7] Brunt D. Supported housing in the community for persons with severe mental illness. Psychosocial environment, needs, quality of life and social network. Lund: Lund University (2002).

[8] Brunt D Rask M. (2018). Resident and staff perceptions of the content of their relationship in supported housing facilities for people with psychiatric disabilities. J Multidiscip Healthc. (2018) 11:673–81. doi: 10.2147/JMDH.S179322, PMID: 30532551 PMC6247964

[9] Sandhu S Priebe S Leavey G Harrison I Krotofil J McPherson P. Intentions and experiences of effective practice in mental health specific supported accommodation services: a qualitative interview study. BMC Health Serv Res. (2017) 17:1–13. doi: 10.1186/s12913-017-2411-0 28693490 PMC5504783

[10] Leamy M Bird V Le Boutillier C Williams J Slade M. Conceptual framework for personal recovery in mental health: systematic review and narrative synthesis. Br J Psychiatry. (2011) 199:445–52. doi: 10.1192/bjp.bp.110.083733, PMID: 22130746

[11] Brolin R Brunt D Rask M Syrén S Sandgren A. Striving for meaning – life in supported housing for people with psychiatric disabilities. Int J Qual Stud Health Well Being. (2016) 11:31249. doi: 10.3402/qhw.v11.31249, PMID: 27172517 PMC4864850

[12] Brolin R Syrén S Rask M Sandgren A Brunt D. Residents’ perceptions of the most positive and negative aspects of the housing situation for people with psychiatric disabilities. Scand J Caring Sci. (2018) 32:603–11. doi: 10.1111/scs.12485, PMID: 28833388

[13] Lindström M. Promoting agency among people with severe psychiatric disability: occupation-oriented interventions in home and community settings. Umeå: Umeå University (2011).

[14] Lindström M Sjöström S Lindberg M. Stories of rediscovering agency: home-based occupational therapy for people with severe psychiatric disability. Qual Health Res. (2013) 23:728–40. doi: 10.1177/1049732313482047 23515296

[15] Killaspy H Priebe S McPherson P Zenasni Z Greenberg L McCrone P. Predictors of moving on from mental health supported accommodation in England: national cohort study. Br J Psychiatry. (2020) 216:331–7. doi: 10.1192/bjp.2019.101 31046864

[16] Bitter NA Roeg DPK van Nieuwenhuizen C van Weeghel J. Identifying profiles of service users in housing services and exploring their quality of life and care needs. BMC Psychiatry. (2016) 16:419. doi: 10.1186/s12888-016-1122-0, PMID: 27881159 PMC5120432

[17] Eklund M Argentzell E Bejerholm U Brunt D Tjornstrand C. Outcomes of the active in my home (AiMH) intervention for people with psychiatric disabilities in supported housing: a longitudinal pilot and feasibility study. Br J Occup Ther. (2019) 83:6–14. doi: 10.1177/0308022619888872

[18] Graneheim U Lundman B. Qualitative content analysis in nursing research: concepts, procedures, and measures to achieve trustworthiness. Nurse Educ Today. (2004) 24:105–12. doi: 10.1016/j.nedt.2003.10.001 14769454

[19] Jose AL Harrison M Roy AS Irvine-Fitzpatrick L Forsyth K. The level of formal support received by people with severe mental illness living in supported accommodation and participation: a systematic review. Int J Soc Psychiatry. (2021) 67:854–66. doi: 10.1177/0020764020988576, PMID: 33487055 PMC8559179

[20] Dalton-Locke C Attard R Killaspy H White S. Predictors of quality of care in mental health supported accommodation services in England: a multiple regression modelling study. BMC Psychiatry. (2018) 18:344. doi: 10.1186/s12888-018-1912-7, PMID: 30342501 PMC6195958

[21] Eklund M Tjörnstrand C. Resident and staff perceptions of an activity and recovery-based intervention in supported housing for people with severe mental illness - a longitudinal pilot study. BMC Psychiatry. (2022) 22:404. doi: 10.1186/s12888-022-04050-7, PMID: 35710347 PMC9205036

[22] Rosenberg D Argentzell E. Service users experience of receiving peer support in Swedish mental health care – a “tipping point” in the care-giving culture? J Psychosoci Rehab Mental Health. (2018) 5:53–61. doi: 10.1007/s40737-018-0109-1

[23] Read J Johnstone L Taitimu M. Psychosis, poverty and ethnicity In: Read J Dillon J, editors. Models of madness: psychological, social and biological approaches to psychosis. 2th ed. London: Brunner-Routledge (2013)

[24] Mattsson M Topor A Cullberg J Forsell Y. Association between financial strain, social network and 5-year recovery from first episode psychosis. Soc Psychiatry Psychiatr Epidemiol. (2008) 43:947–52. doi: 10.1007/s00127-008-0392-3 18604620

[25] Argentzell E Bäckström M Lund K Eklund M. Exploring mediators of the recovery process over time among mental health service users, using a mixed model regression analysis based on cluster RCT data. BMC Psychiatry. (2020) 20:520. doi: 10.1186/s12888-020-02924-2, PMID: 33126873 PMC7602343

[26] Le Boutillier C Leamy M Bird VJ Davidson L Williams J Slade M. What does recovery mean in practice? A qualitative analysis of international recovery-oriented practice guidance. Psychiatr Serv. (2011) 62:1470–6. doi: 10.1176/appi.ps.001312011, PMID: 22193795

[27] Topor A Ljungqvist I. Money, social relationships and the sense of self: the consequences of an improved financial situation for persons suffering from serious mental illness. Community Ment Health J. (2017, 2017) 53:823–31. doi: 10.1007/s10597-017-0146-3, PMID: 28497399 PMC5599438

[28] Pinfold V. Building up safe havens… Around the world': users' experiences of living in the community with mental health problems. Health Place. (2000) 6:201–12. doi: 10.1016/s1353-8292(00)00023-x 10936775

[29] Fossey E Harvey C McDermott F. Housing and support narratives of people experiencing mental health issues: making my place, my home. Front Psych. (2020) 10:939. doi: 10.3389/fpsyt.2019.00939, PMID: 31998158 PMC6966198

[30] Friesinger JG Topor A Bøea TD Larsen IB. Studies regarding supported housing and the built environment for people with mental health problems: a mixed-methods literature review. Health Place. (2019) 57:44–53. doi: 10.1016/j.healthplace.2019.03.006 30959400

[31] Bitter N Roeg D van Nieuwenhuizen C van Weeghel J. Recovery in supported accommodations: a scoping review and synthesis of interventions for people with severe mental illness. Community Ment Health J. (2020) 56:1053–76. doi: 10.1007/s10597-020-00561-3, PMID: 32016620 PMC7289772

[32] Cook S Hill C Mundy T Killaspy H Holloway F Craig T. GetREAL intervention manual. A staff training intervention for inpatient mental health rehabilitation units aimed at increasing patients’ engagement in activities. Sheffield: Sheffield Hallam University (2012).

[33] McPherson P Lloyd-Evans B Dalton-Locke C Killaspy H. A systematic review of the characteristics and efficacy of recovery training for mental health staff: implications for supported accommodation services. Front Psych. (2021) 12:624081. doi: 10.3389/fpsyt.2021.624081, PMID: 34054593 PMC8160251

[34] Nilsson I Townsend E. Occupational justice-bridging theory and practice. Scand J Occup Ther. (2010) 17:57–63. doi: 10.3109/11038120903287182, PMID: 20170412

[35] Lincoln YS., & Guba EG. (1985). Naturalistic inquiry. Beverly Hills, CA: Sage Publications, 9, 438–439.

